# Effect of Early Vestibular Rehabilitation on Vertigo and Unsteadiness in Patients with Acute and Sub-Acute Head Trauma

**Published:** 2018-03

**Authors:** Sadegh Jafarzadeh, Akram Pourbakht, Eshagh Bahrami, Shohreh Jalaie, Arash Bayat

**Affiliations:** 1 *Department of Audiology, School of Paramedical Sciences, Mashhad University of Medical Sciences, Mashhad, Iran.*; 2 *Department of Audiology, School of Rehabilitation Sciences* *, Iran University of Medical Sciences, Tehran, Iran.*; 3 *Department of Neurosurgery, Iran University of Medical Sciences, Tehran, Iran.*; 4 *Department of Biostatistics, School of Rehabilitation, Tehran University of Medical Sciences, Tehran, Iran. *; 5 *Hearing Research Center, Imam Khomeini hospital, Ahvaz Jundishapur University of Medical Sciences, Ahvaz, Iran. *

**Keywords:** Dizziness Handicap Inventory, Head trauma, Vestibular rehabilitation, Unsteadiness, Vertigo

## Abstract

**Introduction:**

Vestibular rehabilitation is a treatment option for the management of vertigo and unsteadiness, which are very common in head trauma patients and more challenging in the early months after trauma. This study evaluated the effectiveness of a vestibular rehabilitation program in the recovery of acute and sub-acute head trauma patients. The goal of this study was evaluation of the effect of early vestibular rehabilitation on patients with acute and sub-acute head trauma.

**Materials and Methods:**

This study was performed in 20 head trauma patients with vertigo and unsteadiness. The patients were randomly divided into two groups: one group received medical therapy (Betaserc) and the other received rehabilitation and medical therapy. An individualized vestibular rehabilitation program was designed that was then revised and verified by a joint committee of vestibular rehabilitation groups. The effectiveness of interventions was measured using the Dizziness Handicap Inventory (DHI) by comparing the results before and after therapy.

**Results:**

The physical conditions and DHI scores of patients in both groups were similar at baseline. After 1 month of rehabilitation, patients receiving vestibular rehabilitation and medication showed greater progress than patients receiving medication only (P=0.000).

**Conclusion:**

Vestibular rehabilitation can aid in the recovery from vertigo and increase the stability of head trauma patients. Simultaneous treatment with medicine and vestibular rehabilitation exercises can result in quicker and better therapeutic effects.

## Introduction

Head trauma can be caused by any incident such as falling, an accident at work or a quarrel. It is a common event, and the associated symptoms of vertigo and unsteadiness are prevalent in head trauma patients (1,2). The vestibular assessment of head trauma patients shows a high rate of vestibular lesions , especially in the peripheral vestibular system (2,3). Different vestibular disorder scan be caused by head trauma, including benign paroxysmal positional vertigo, labyrinthine concussion, rupture of the round window membrane, perilymphatic fistula and endolymphatic hydrops, although several patients experiencing vertigo do not have any of these disorders (1,4-6). Head trauma may also cause abnormalities in the otolith system or other structures that cause vertigo and unsteadiness (1,5,7,8).

Vestibular rehabilitation is a treatment option for the management of vertigo and unsteadiness that could result in the recovery or improvement of symptoms. 

In most cases, because of the patient’s physical and emotional condition, vestibular rehabilitation is initiated in the chronic stage of the disease, and it can be very challenging in the acute stage. 

Despite the large number of patients and the damaging consequences of vertigo and unsteadiness, only a few studies have assessed the effect of vestibular rehabilitation in acute and sub-acute head trauma patients (up to 6 months after head trauma). These few studies have suggested that early rehabilitation can obviate vertigo associated with head trauma and be beneficial in head trauma patients (2). The most common vestibular tests, such as the caloric test, can assess the peripheral vestibular system but are not able to show patient recovery, and results remain abnormal even after successful recovery (9). 

For this reason, a questionnaire is commonly used for the assessment of patients before and after therapy.Vestibular rehabilitation is usually performed at the chronic stage of head trauma, although it would actually be helpful in the acute stage. 

Therefore, the objective of the present study was to evaluate the effect of early vestibular rehabilitation in patients with acute and sub-acute head trauma using DHI.

## Materials and Methods

*Participants*

This study was performed from March 2013 to January 2014 in 20 adult patients (aged 18–60 years) referred to a vestibular rehabilitation center by ear-nose-throat or neurosurgery specialists. All patients had undergone complete neurologic and otologic evaluations and hospital discharge, and had normal computed tomography (CT) scans and an absence of physical abnormalities including benign paroxysmal positional vertigo, rupture of the round window membrane, perilymphatic fistula and endolymphatic hydrops. Patients showed no remarkable pathology (e.g., fractures, wounds, intracranial pathology or neurological deficits).

A hearing assessment revealed five patients with normal hearing, eight with bilateral and slight sensorineural hearing loss (SNHL) at high frequencies that could be related to their age, two with general bilateral and slight SNHL, three with bilateral moderate SNHL, one with bilateral profound SNHL and one with unilateral moderate SNHL. No patients with conductive or mixed hearing loss were enrolled into the study. The SNHL was not permitted to affect the vestibular tests including cervical vestibular evoked myogenic potential (cVEMP), but was required to show damage to the auditory system. The inclusion criteria were vertigo and unsteadiness due to head trauma that was caused by falling or an accident in the past 6-months; lack of cognitive, cervical, visual and neck problems in the neurologic and otologic evaluations; Glasgow coma score higher than 9 (10); and abnormal results in cVEMP or Dynamic Posturography indicating a vestibular abnormality.

Patients who met the study criteria were randomly classified into two groups. All patients were informed about the study in detail and their consent was obtained for participation in the study. This study was approved by the Ethics Committee of our local university (91d1303430).

*Procedures*

Vestibular assessment: Vestibular assessment of the patients was performed at the beginning of the program, including a case history, bedside examination, assessment of spontaneous nystagmus, horizontal and vertical gaze and saccade, Romberg’s test, Dix–Hallpike maneuver, cVEMP (Labat, Italy) and Dynamic Posturography (Equitest, USA). These tests were used to determine the site of the lesion of the vestibular disorder and to confirm inclusion criteria. The Persian version of the DHI was used to measure the physical, emotional and functional outcomes of head trauma and the progress during and after 4 weeks of vestibular rehabilitation (11).

cVEMP: cVEMPs were recorded ipsilaterally from the sternocleidomastoid (SCM) muscle using acoustic stimulation. Tone bursts stimuli (500 Hz, 5/s) were presented monaurally through headphones. The active, reference and ground electrodes were located over the middle of the SCM, upper sternum and forehead, respectively. The electrode impedance was maintained under 3 kΩ. Subjects turned their head to the contralateral shoulder before starting the measurement and held this position constantly to achieve a constant tonic activation of the SCM during the recording period. The tonic activation also was monitored.

Dynamic posturography: This test was performed under the usual six conditions of the sensory organization test, and the results of positions 5 and 6 were used for detecting vestibular abnormalities as inclusion criteria.

DHI: DHI is a valuable tool for assessing the patient’s recovery during the follow-up period. The DHI is a valid and reliable questionnaire that measures the effect of vertigo and unsteadiness on different physical (9), emotional and functional aspects of a patient’s life (9), quality of life and treatment progress (12). The DHI has a widespread use in research and clinical settings. The DHI consists of 25 questions divided into three areas of a patient’s life: physical (seven questions), functional (nine questions) and emotional (nine questions). Each item is assigned 0, 2, or 4 points, with the total DHI score ranging from 0 (no impairment) to 100 (severe impairment) points (9). In this study, both the total DHI score and the score for each domain were calculated. The DHI score was classified as no activity limitation or participation restriction for scores between 0 and 14, and as mild, moderate and severe impairment for scores of 16–26, 28–44 and 46–100 points, respectively (13).Romberg’s test: Patients were asked to stand with their feet together and open and then close their eyes. The examiner observed how patients were able to maintain their balance in an upright posture.

*Intervention*

After the vestibular assessment, participants were randomly divided into two groups. The first group received the usual medical therapy (Betaserc 8 mg pills; at least three pills per day), and the second group received medical therapy and vestibular rehabilitation.

The vestibular rehabilitation program was designed for daily training for a 4-week period and included mostly gaze stability and adaptation exercises, and some substitution exercises (Table.1). 

**Table 1:** Most important exercises used by patients

**Gaze stability and adaptation**	**Substitution**
Near object, horizontal X1	Moving head right and left and up and down
Near object, vertical X1	Standing, open and closed eye
Distant object, horizontal X1	Standing, Moving head, horizontally and vertically
Distant object, vertical X1	Leaning to left and right side
Near huge object, horizontal X1	Standing on soft platform
Near huge object, vertical X1	Walking with head movement
Near object, horizontal X2	Walking, feet hold closer together
Near object, vertical X2	Walking on a ramp
Near huge object, horizontal X2	Catching and throwing a ball
Near huge object, vertical X2	Walking on a soft platform
Looking a ball in air and passing it between two hands	Bouncing on a big ball

These exercises were selected for each patient based on their function and vestibular assessment test results. Different gaze stability and adaptation exercises were used in all patients, although substitution exercises including standing and walking exercise were used only in patients with unsteadiness. The vestibular rehabilitation program was revised and verified by the joint committee of vestibular rehabilitation groups at our local university. These exercises were performed by therapists with specialized training in the assessment and treatment of vestibular disorders.

*Data analysis*

Data were analyzed using SPSS version 19.0 computer software. A p-value below 0.05 was considered statistically significant. Descriptive analysis (mean and standard deviation) included demographic factors such as age, sex and duration of symptoms. The DHI was the only assessment that was repeated every week, and its scores were compared with repeated measures analysis of variance (ANOVA) at baseline and during and after vestibular rehabilitation in the two groups.

## Results

Patients had a mean age of 44.2 (standard deviation [SD]: 12.6) years. Table 2 shows the total and subtest DHI scores at the beginning of the vestibular rehabilitation program. We compared each subtest and total score of DHI between the two groups to check for equality in the two groups at the start of the study. There was no significant difference between the two groups in the total score and subtests at the beginning of the program (Table. 2).

**Table 2:** Total and subtest scores (mean and standard deviation) of DHI at the beginning of the vestibular rehabilitation program

**Group**	**Functional**	**Emotional**	**Physical**	**Total score**
1	13.4 (9.2)	9.4 (6.2)	8.2 (9.8)	31.0 (24.3)
2	17.2 (9.1)	12.0 (11.2)	13.8 (8.8)	43.0 (25.4)
P-value	0.366	0.532	0.197	0.295

Group 1= Medical therapy

Group 2= Medical therapy and vestibular rehabilitation

Table 3 shows the recovery status during the vestibular rehabilitation program during weeks 1–4 in the two groups. There were no significant differences between the two groups at weeks 1 and 2, but significant differences were observed at weeks 3 and 4. The progress and improvement of DHI scores represent recovery and improvement in quality of life. In addition, the Romberg’s tests were improved in some patients who received vestibular rehabilitation.

**Table 3:** Change between first and subsequent total DHI scores (recovery) during the vestibular rehabilitation period (week 1 to 4) in the two groups

**Group**	**Week**			
	**1** **Mean (SD)**	**2** **Mean(SD)**	**3** **Mean(SD)**	**4** **Mean (SD)**
1	1.8 (10.9)	0.6 (3.8)	0.4 (6.0)	0.2 (7.8)
2	−2.0 (8.7)	3.2 (11.2)	16.4 (8.8)	20.0 (11.0)
P-value	0.657	0.498	0.000*	0.000*

Group 1= Medical therapy Group 2= Medical therapy and vestibular rehabilitation

*P-value is significant at 0.001 level

Table 4 shows the changes in the different subtests of DHI in the two groups. The participants in the medical therapy and vestibular rehabilitation group had a higher change in functional and physical subtests.

**Table 4:** Changes in DHI scores after 4 weeks

**Group**	**DHI-P**		
	**Functional**	**Emotional**	**Physical**
1	−0.2 (4.1)	−0.4 (2.4)	0.8 (2.5)
2	8.0 (6.0)	4.4 (4.9)	7.6 (6.2)

Group 1= Medical therapy

Group 2= Medical therapy and vestibular rehabilitation

## Discussion

Vestibular rehabilitation is a procedure that can be used for the treatment of vertigo and unsteadiness (14). Owing its success, there has been an increasing interest in this procedure for use in patients with vestibular disorders (15). 

In the present study, we used an individualized vestibular rehabilitation program for head trauma patients with vertigo and unsteadiness. The results show that an early vestibular rehabilitation program within a time period of 1 month can decrease vertigo symptoms and increase stability and balance performance, thereby improving the patient’s functional and physical conditions.

Our results show that early vestibular rehabilitation could resolve the patient’s problem, or at least decrease it. Similar studies have also reported the ability of vestibular rehabilitation to decrease the harmful effect of vertigo and unsteadiness (2,5,16–19). It has been observed that early rehabilitation can be useful and short-term rehabilitation can decrease the consequences of head trauma (2,16), although a few poor results were also observed in some cases (5).

Although clinical experience shows that medications can help some patients to resolve their symptoms, the control group in our study showed only a modest change during the 1-month follow-up. This could be due to the fact that this follow-up period is a short time for observing the therapeutic effects. Therefore, simultaneous treatment with medicine and vestibular rehabilitation exercises can have a quicker and better therapeutic effect, at least during the early treatment phase.

Most of our study participants who underwent rehabilitation had high DHI scores; however, after completion of the program, most were symptom free or experiencing a mild level of disability. Decreasing the severity of disability leads to an independent life and increases the patient’s quality of life (15). According to the DHI score classification (13), six patients with severe, four with moderate, eight with mild and two with no reported handicap entered our study (two patients with severe, two with moderate, four with mild and two with no reported handicap in the medical therapy group, and four patients with severe, two with moderate, four with mild and no patients with no reported handicap in the medical therapy and vestibular rehabilitation group). At the end of the 4-week program, there was no change in the classification of participants in the medical therapy group and none of them had any change beyond the smallest detectable change in DHI value (11); however, six out of ten participants in the medical therapy and vestibular rehabilitation group had a change beyond that point and eight out of ten had sufficient changes to be placed in lower classifications.

Vestibular rehabilitation can decrease the functional, emotional and physical consequences of vertigo and unsteadiness and improve the patients’ quality of life. In our study, the functional and physical subtests of DHI showed a higher change than the emotional subtest. It is possible that changing the emotional status requires more time and psychological support.

The strength of the present study lies in using a control group to better describe the vestibular rehabilitation effect and begin the rehabilitation program in the short period after the head trauma event. It has been reported that a long period between the head trauma and onset of rehabilitation can decrease the effectiveness of the procedure (5), and using the short-term and individualized training can improve the patients’ condition (16). However, a limitation of the study was its short follow-up time. In order to fully understand the vestibular rehabilitation effect, a follow-up time of 3 to 6 months would be required.

We used an individualized vestibular rehabilitation program that is based on the patient’s problem, site of lesion and the patient’s functional status. Different treatment strategies would be necessary for different types of abnormalities in vestibular mechanisms (20). Individualized vestibular exercises obtain better outcomes for patients (21); hence, the therapist must provide an individualized program for each patient and use proper training for patients with special needs or depending on the site of the lesion. For instance, using auditory feedback with vestibular training resulted in good therapeutic effects in patients with otolith abnormalities (16,22).

## Conclusion

Vestibular rehabilitation can aid in the recovery from vertigo and increase the stability of head trauma patients. Simultaneous treatment with medicine and vestibular rehabilitation exercises can result in quicker and better therapeutic effects.
